# The effects of partial wrist denervation in wrist osteoarthritis: patient-reported outcomes and objective function

**DOI:** 10.1177/17531934221088498

**Published:** 2022-03-28

**Authors:** Elin M. Swärd, Mikael Andersson Franko, Maria K. Wilcke

**Affiliations:** 1 Karolinska Institutet, Department of Clinical Science and Education, Södersjukhuset, Stockholm, Sweden; 2 Department for Hand Surgery Södersjukhuset, Stockholm, Sweden

**Keywords:** Wrist denervation, patient-reported outcomes, Patient-Rated Wrist Evaluation, Disabilities of the Arm, Shoulder and Hand, Quality of life, arthritis

## Abstract

This prospective longitudinal study aimed to analyse the effect of partial wrist denervation on patient-reported outcomes, quality of life and objective function in symptomatic wrist osteoarthritis during the first year after surgery. Sixty consecutive patients underwent an anterior and posterior interosseous neurectomy during 2018–2020. Disabilities of the Arm, Shoulder and Hand, Patient-Rated Wrist Evaluation, EuroQol-5D-3L, pain at rest and on load, and objective function were assessed preoperatively and 3, 6 and 12 months postoperatively. Generalized estimating equations were used to analyse the effect on the outcome variables. Disabilities of the Arm, Shoulder and Hand, Patient-Rated Wrist Evaluation and pain scores improved significantly postoperatively with no decline over time, but no patient reported outcome measure reached the minimal clinically important difference. Quality of life, strength and range of motion did not improve. We found no complications. Seventeen patients needed further surgery during the study period. More studies are needed to evaluate whether denervation is truly effective or not.

**Level of evidence:** II

## Introduction

Wrist denervation for the management of chronic pain was described by Wilhelm in the 1960s (Wilhelm, 1965; Wilhelm, 1966). Denervation aims to reduce pain by transection of peripheral sensory nerve branches that exclusively innervate the wrist joint capsule. A complete wrist denervation as described by Wilhelm requires multiple incisions and extensive dissection, and subsequently partial denervation techniques have been developed to reduce the surgical trauma (Berger, 1998). Symptom relief has been reported after isolated neurectomy of the anterior interosseous nerve (AIN) (Dellon et al., 1984) or posterior interosseous nerve (PIN) (Dellon, 1985; Ferreres et al., 1995; Riches et al., 2014) and after a combined AIN and PIN neurectomy through a single dorsal skin incision (Berger, 1998; Hofmeister et al., 2006; O'Shaughnessy et al., 2019; Weinstein and Berger, 2002).

Partial wrist denervation is mainly used in the treatment of painful osteoarthritis, but also in the treatment of chronic pain secondary to carpal instability, scaphoid nonunion, after fractures and inflammatory arthritis (Kadhum et al., 2020). In contrast to partial wrist fusions and proximal row carpectomy, denervation does not require immobilization, is technically easier, preserves motion and does not preclude future salvage procedures. Also, the risk of complications is lower (Kadhum et al., 2020; Wysocki and Cohen, 2010). Although widely used, evidence regarding the effect of partial wrist denervation is limited. Most previous studies are small, and few studies employ standardized follow-up protocols at regular intervals or use validated patient-reported outcome measures (PROMs) before and after surgery (Chin et al., 2020; Kadhum et al., 2020; Smeraglia et al., 2020).

The primary aim of this study was to assess the effect of partial wrist denervation for painful wrist osteoarthritis on the Disabilities of the Arm, Shoulder and Hand (DASH) (Hudak et al., 1996) score during the first year after surgery. Secondary aims were to assess changes in the Patient-Reported Wrist Evaluation (PRWE) (MacDermid et al., 1998) score, pain at rest and on load assessed by a Numerical Rating Scale (NRS), European Quality of Life Five Dimension (EuroQol-5D-3L (EQ5D-3L)) (Brooks, 1996) and objective function (range of movement (ROM), grip strength, key pinch). We hypothesized that partial denervation would improve PROMs, quality of life and objective function.

## Methods

### Surgical technique

**Figure 1.**
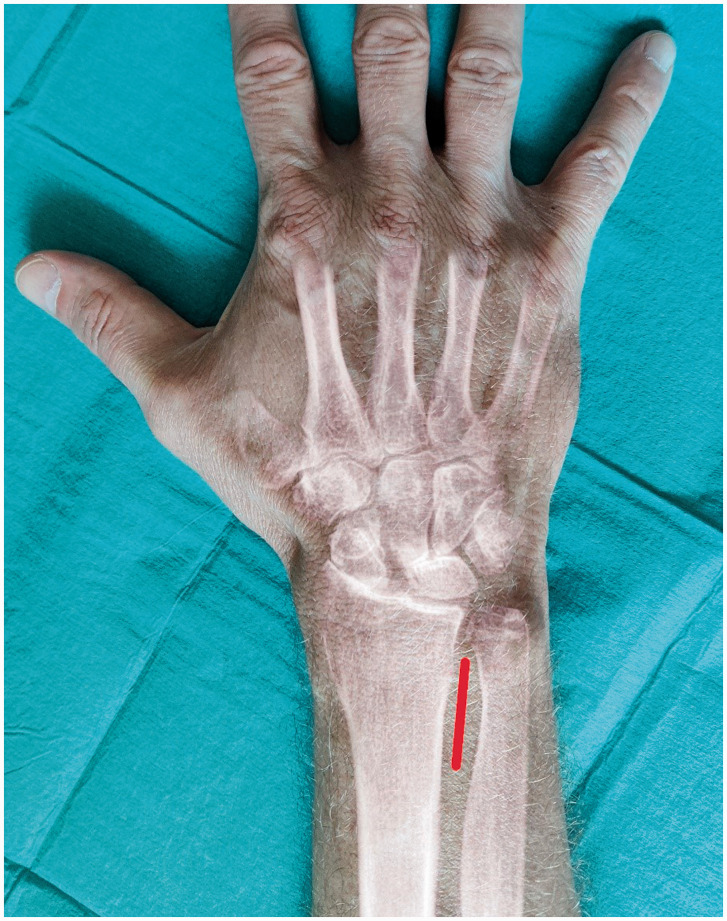
Illustration of the surgical approach for wrist denervation. Red line indicates the position of skin incision just proximal to the distal radioulnar joint (DRU).

In this prospective longitudinal study, 60 consecutive patients underwent combined AIN and PIN neurectomy through a single dorsal approach as described by Berger (1998) (Figure 1) at the Department of Hand Surgery, Södersjukhuset, Stockholm, between January 2018 and August 2020. Two centimetres of the PIN were resected. After incision of the interosseous membrane, the AIN was traced distally to within 2 cm of the ulnar head, and 1 cm was resected at this level to ensure that the motor branches innervating the pronator quadratus were protected. Operations were performed by surgeons with Level III expertise (Tang and Giddins, 2016) in day surgery under axillary block or local anaesthesia. A soft dressing was used, and active motion was started immediately postoperatively. The patients received oral and written instructions regarding mobilization. No formal hand therapy protocol was used. Sutures were removed at the clinic after 2 weeks.

### Inclusion and exclusion criteria

The inclusion criteria were symptomatic wrist osteoarthritis caused by scapholunate advanced collapse (SLAC), scaphoid nonunion advanced collapse (SNAC), osteoarthritis after distal radial fracture or osteoarthritis secondary to Kienböck’s disease (Table 1). Only patients who had tried non-surgical management, such as bracing, corticosteroid injections, oral analgesics and/or physiotherapy, were included.

**Table 1.** Types of wrist osteoarthritis.

Diagnosis	Number of patients
SLAC1	4
SLAC2	18
SLAC3	21
SNAC1	4
SNAC2	2
SNAC3	5
Osteoarthritis after distal radial fracture	4
Kienböck’s disease (Stage 4)	2

SLAC: scapholunate advanced collapse, Stage 1–3 according to the classification by Watson and Ballet (1984); SNAC: scaphoid nonunion advanced collapse, Stage 1–3 according to the classification by Vender et al. (1987).

The exclusion criteria were age <18 years, rheumatoid or other inflammatory arthritis, symptomatic idiopathic osteoarthritis, previous PIN or AIN neurectomy on the ipsilateral side and symptomatic instability of the distal radioulnar (DRU) joint on the ipsilateral side. Patients who were unable to cooperate with the follow-up protocol due to language difficulties, severe psychiatric disorder, cognitive impairment or drug addiction were also excluded.

### Outcome assessments

The primary outcome variable was change in DASH score (0–100 points) measured before surgery and 3, 6 and 12 months postoperatively. Secondary outcome variables, measured at the same timepoints, were changes in PRWE (0–100), EQ5D-3L (0–1), pain at rest and on load (NRS, 0–10), ROM, key pinch, grip strength, complications related to surgery and need for a secondary salvage procedure. Based on previous research, the minimal clinical important differences (MCIDs) were set to 10 for DASH (Sorensen et al., 2013), 14 for PRWE (Sorensen et al., 2013), 0.1 for EQ5D-3L (Walters and Brazier, 2005) and two for NRS (Salaffi et al., 2004). Key pinch was measured with a pinch gauge (PG-30, B&L Engineering®, Santa Ana, CA, USA) and grip strength with a hydraulic hand dynamometer (BL5001, B&L Engineering®, Santa Ana, CA, USA) and recorded as the mean of three attempts at maximal grip. ROM was measured using a goniometer with 5° intervals. The PROMs were sent to research subjects by mail prior to the visits.

### Statistical analysis

MCID of the primary outcome variable, DASH score, was set at 10 points based on previous reports (Sorensen et al., 2013). A sample size of 50 was required to detect this change presuming a SD of 20 and correlation coefficient (r) = 0.20. Sixty patients were included to account for some loss to follow-up. For descriptive statistics, DASH, PRWE, EQ5D-3L and pain NRS scores were reported as median (interquartile range (IQR)) and continuous variables as mean (standard deviation (SD)). To analyse the repeated measurements, we used generalized estimating equations (GEE) with robust estimator covariances matrix, independent working correlation matrix (according to lowest Quasi-likelihood under Independence Model Criterion (QICC)) and a linear model for all variables. High preoperative pain level at rest (defined as above the 75th percentile), sex, age ≥65 and operated dominant hand were included as possible predictors in the model. The changes in outcome variables over time and the effects of the predictors are presented as beta coefficient (β) with *p*-value. β is the expected population average change of the outcome variable between the preoperative values and the assessments 3, 6 and 12 months after operation. Significance was set at *p* ≤ 0.05.

## Results

One patient was lost to follow-up 6 months postoperatively due to illness unrelated to the denervation surgery. Due to the COVID-19 pandemic, five patients declined clinical assessment and therefore their objective physical variables are missing at one timepoint. Completeness of the DASH and PRWE scores, was 98% and 99%, respectively. The mean age at the time of surgery was 60 years (range 24–87). Forty-three of the patients were men (72%). The dominant side was affected in 42 wrists (70%). Thirty-two patients were manual labourers, seven had non-manual work and 21 were retired. Fifteen patients had had one operation on the ipsilateral wrist prior to the denervation surgery, including diagnostic arthroscopy (*n* = 5), distal radial fracture fixation (*n* = 4), ganglion excision (*n* = 4) and scaphoid nonunion surgery (*n* = 2).

The values of the outcome variables before and at each assessment after operation are presented in Table 2. Pain at rest and on load, DASH and PRWE all improved significantly after denervation, but the improvement did not reach the MCID level (Table 3). Denervation had no significant effect on EQ5D-3L. A high preoperative pain level (>6 NRS) at rest predicted more pain at rest and on load (β = 3 (*p* < 0.001) and β = 2 (*p* < 0.001), respectively) and a worse DASH, PRWE and EQ5D-3L-score (β = 16 (*p* = 0.002), β = 18 (*p* < 0.001) and β = −0,30 (*p* < 0.001), respectively) at follow-up.

**Table 2.** PROMs, strength, and range of motion before and after wrist denervation.

Measurement	Preoperatively	Postoperatively		
		3 months	6 months	12 months
DASH (0–100)	45 (28–58)	40 (26–55)	35 (24–49)	37 (24–49)
PRWE (0–100)	63 (53–75)	57 (43–70)	59 (43–71)	57 (42–68)
EQ5D-3L (0–1)	0.69 (0.23–0.78)	0.71 (0.51–0.80)	0.69 (0.55–0.76)	0.73 (0.52–0.80)
NRS at rest (0–10)	5 (2–6)	3 (2–5)	3 (2–4)	3 (1–5)
NRS on load (0–10)	8 (6–9)	7 (4–8)	7 (5–8)	7 (5–8)
Grip strength (kg)	21 (11)	22 (12)	21 (12)	23 (12)
Pinch strength (kg)	6.4 (2.6)	6.2 (2.7)	6.1 (2.8)	6.5 (2.7)
Extension (°)	41 (14)	43 (13)	43 (13)	40 (16)
Flexion (°)	37 (17)	39 (13)	39 (12)	39 (13)
Ulnar deviation (°)	24 (8)	25 (11)	26 (9)	27 (11)
Radial deviation (°)	13 (7)	12 (9)	12 (6)	10 (7)
Supination (°)	80 (8)	71 (16)	72 (11)	73 (10)
Pronation (°)	75 (11)	72 (12)	74 (7.9)	72 (7.5)

DASH, PRWE, EQ5D-3L and NRS presented as median (IQR). Grip strength, key pinch and range of movement presented as mean (SD).

DASH: Disabilities of the Arm, Shoulder and Hand; PRWE: Patient-Rated Wrist Evaluation; EQ5D: EuroQol-5D-3L; NRS: Numerical Rating Scale.

**Table 3.** Effect of wrist denervation on PROMs, strength and range of motion.

Measurement	Preoperatively – 3 months	Preoperatively – 6 months	Preoperatively – 12 months
DASH^a^	−3.6 (*p* = 0.04)	−4.3 (*p* = 0.02)	−4.8 (*p* = 0.03)
PRWE^a^	−6.8 (*p* < 0.01)	−5.0 (*p* = 0.04)	−8.3 (*p* = 0.01)
EQ5D-3L^a^	0.054 (*p* = 0.12)	0.040 (*p* = 0.22)	0.081 (*p* = 0.06)
NRS at rest^a^	−1.0 (*p* < 0.01)	−0.9 (*p* < 0.01)	−0.8 (*p* = 0.03)
NRS on load^a^	−0.9 (*p* < 0.01)	−0.5 (*p* = 0.11)	−1.0 (*p* = 0.01)
Grip strength^a^	0.5 (*p* = 0.55)	0.5 (*p* = 0.61)	1.6 (*p* = 0.15)
Pinch strength^a^	−0.3 (*p* = 0.14)	−0.2 (*p* = 0.32)	0.1 (*p* = 0.54)
Extension^a^	1.8 (*p* = 0.30)	2.3 (*p* = 0.26)	−0.7 (*p* = 0.76)
Flexion^a^	1.5 (*p* = 0.43)	1.4 (*p* = 0.51)	1.9 (*p* = 0.39)
Ulnar deviation^a^	1.3 (*p* = 0.37)	2.2 (*p* = 0.13)	2.7 (*p* = 0.08)
Radial deviation^a^	−0.4 (*p* = 0.77)	−0.9 (*p* = 0.44)	−2.4 (*p* = 0.05)
Supination^a^	−8.6 (*p* < 0.01)	−7.5 (*p* < 0.01)	−6.2 (*p* < 0.01)
Pronation^a^	−3.2 (*p* = 0.05)	−1.0 (*p* = 0.39)	−2.9 (*p* = 0.03)

Results expressed as beta coefficient: expected population average change of the outcome variable between the assessments (*p*-value)

a Corrected for age ≥65, NRS pain at rest >6, gender, operated dominant hand.

DASH: Disabilities of the Arm, Shoulder and Hand; PRWE: Patient-Rated Wrist Evaluation; EQ5D: EuroQol-5D; NRS: Numerical Rating Scale.

There were no significant changes in grip strength or key pinch at any of the assessments and no improvement in range of motion after the denervation. Supination, pronation and radial deviation had deteriorated significantly at 12 months postoperatively. However, the differences were small. There was an improvement in DASH, PRWE and pain at rest and on load above MCID in roughly one-third of the patients (Table 4). Logistic regression analyses could not identify any significant predictors for improvement above MCID level (online supplemental material, Table S1).

**Table 4.** Changes in PROMs in relation to MCID-levels 12 months postoperatively.

PROM	Change (points)	Number of patients
DASH	≥10 improvement	20
	≥10 deterioration	7
	Decrease or increase smaller than MCID	24
PRWE	≥14 improvement	19
	≥14 deterioration	7
	Decrease or increase smaller than MCID	25
EQ5D-3L	≥0.1 improvement	16
	≥0.1 deterioration	10
	Decrease or increase smaller than MCID	25
NRS at rest	≥2 improvement	20
	≥2 deterioration	10
	Decrease or increase smaller than MCID	22
NRS on load	≥2 improvement	19
	≥2 deterioration	8
	Decrease or increase smaller than MCID	25

DASH: Disabilities of the Arm, Shoulder and Hand (0–100, MCID 10); PRWE: Patient-Rated Wrist Evaluation (0–100, MCID 14); EQ5D-3L: EuroQol-5D-3L (0–1, MCID 0.1) NRS for pain: Numerical rating scale (0–10, MCID 2); MCID: minimal clinically important difference.

No postoperative complications were noted. Seven patients needed further wrist surgery within 12 months after operation and ten patients were scheduled for a subsequent salvage surgery at the 12-month follow-up visit. These cases were considered as failures, leading to a failure rate of 28%. Of the failures, 15 underwent scaphoid excision and partial wrist fusion, one a proximal row carpectomy and one (a SLAC1 wrist) a wrist capsulodesis. Men and manual labourers were more likely to need subsequent salvage surgery during the study period compared with women and office workers or retired patients (Table S2).

## Discussion

We found improvement in PROMs after partial wrist denervation for painful wrist osteoarthritis but no improvement in quality of life, strength or range of motion. Although the improvements in PROMs were statistically significant, they were below the MCID (Salaffi et al., 2004; Sorensen et al., 2013; Walters and Brazier, 2005), and therefore it is questionable whether these changes are clinically relevant. There have been concerns that denervation of the PIN may alter proprioceptive reflexes of the wrist (Hagert and Persson, 2010), but in a recent study of proprioception after complete wrist denervation, no changes in the proprioceptive sense of joint position, reflex time or force sense were found (Rein et al., 2020).

The level of evidence is low regarding the effect of different types of wrist denervation and meta-analyses have been impossible to perform due to heterogeneity of the reported outcome variables (Chin et al., 2020; Kadhum et al., 2020; Smeraglia et al., 2020). The improvements in PROMs in this study are considerably smaller than previous reports on partial wrist denervation (Abdelaziz et al., 2019; Hofmeister et al., 2006; O'Shaughnessy et al., 2019; Riches et al., 2014). Comparison between previous studies and ours is complicated by different indications for surgery, diverse PROM scales and the fact that none of these studies reported PROMs at specified timepoints postoperatively, only at last follow-up visit, which varied considerably in time both within and between studies. Hofmeister et al. (2006) investigated patients with wrist instability and reported a mean DASH improvement of 15 points in 50 wrists 24–42 months after AIN and PIN resection. Abdelaziz et al. (2019) reported an average improvement of 15 points in DASH score 12–30 months after PIN denervation in 30 wrists with SLAC/SNAC osteoarthritis. Riches et al. (2014) reported an average PRWE improvement from 86.6 to 43 points 3–48 months after PIN resection in 14 wrists with rheumatoid arthritis. Some of the preoperative items of the PRWE scores were collected retrospectively, leading to a risk of recall bias. O’Shaugnessy et al. (2020) included wrists with both osteoarthritis and inflammatory arthritis and found an average improvement in Mayo Wrist Score (MWS, 0–100 points) from 48 to 77, 1–21 years after PIN and AIN denervation in 100 wrists. Postoperative MWS was only available in 61% of patients, which may have influenced the results.

There are several possible reasons for the difference between our results and previous studies. Instability may cause temporary inflammatory pain and pain due to rheumatoid arthritis may fluctuate with time and medication. Hence, there is a risk that improvements formerly reported may be overrated. Also, earlier studies have not adjusted the results for confounding factors. Finally, the previously reported effects could be due to placebo or chance regression to the mean. Placebo is reported to be effective in the treatment of osteoarthritis, especially for pain, stiffness and self-reported function. Also, the placebo effect increases with increased baseline pain severity (Zhang et al., 2008). In this study, we found that a high preoperative pain score had an impact on all PROMs. Consequently, it is a strength of our study that pain level before surgery is corrected for in the analysis.

Our finding that partial denervation does not improve ROM is in accordance with previous reports (Abdelaziz et al., 2019; Hofmeister et al., 2006; Sgromolo et al., 2018). We found a small deterioration of rotation and radial deviation. This could reflect a natural deterioration of the osteoarthritic joint with progressive decline of movement. However, the change was small, and we do not consider it as clinically relevant, especially considering the possible measurement error. We found no improvement neither in grip strength nor pinch. This is in contrast with previous studies that have reported significant 16–34% improvement in grip strength (Abdelaziz et al., 2019; Hofmeister et al., 2006; Weinstein and Berger, 2002).

The rate of requirement for further surgery in our study (28% after 12 months postoperatively) is comparable with the revision rate reported by Kadhum et al. (2020) (24% 18 months postoperatively). Our finding that men and manual workers were more likely to need revision surgery suggests that these patients may benefit from more extensive salvage procedures, such as proximal row carpectomy or four-corner fusion, rather than partial wrist denervation.

The PIN and AIN innervate two-thirds of the central part of the wrist joint (Berger, 1998). Hence, PIN and AIN denervation may not be sufficient in SLAC/SNAC osteoarthritis, where the pathological changes, even in early stages of disease, are located at the radial parts of the radiocarpal joint. This theory is supported by previous findings of lower revision rate among patients with non-SNAC/SLAC osteoarthritis (O'Shaughnessy et al., 2019). Consequently, the smaller improvement in DASH and PRWE in our study, may partly be explained by the high proportion (90%) of SLAC/SNAC.

Like previous studies, our study has methodological flaws. The major limitations are the lack of control group and blinding, leading to a risk for bias and confounding. Potential confounders are included in the GEE model to minimize this risk. Other limitations include a relatively short follow-up time and that the underlying causes of wrist osteoarthritis are heterogenous, which may potentially influence the result. The foremost strengths of this study are a relatively large sample size of 60 consecutive patients collected during a short time span, the use of validated and widely used PROMs, a prospective design, a structured follow-up protocol and a low dropout rate.

In summary, we found that for painful wrist osteoarthritis, partial denervation had a small but significant effect on patient-reported outcomes 1 year after surgery. One-third of the patients improved above MCID in all PROMs, but the population average improvement in PROMs did not reach MCID. We could not identify any specific patient characteristics that predicted a better outcome. Consequently, it is uncertain if partial wrist denervation has a clinical effect. Perhaps specific patient groups may benefit, but to evaluate if wrist denervation is truly effective, randomized controlled trials are essential.

## Trial registration

ClinicalTrials.gov: NCT03378362.
